# Microglia display modest phagocytic capacity for extracellular tau oligomers

**DOI:** 10.1186/s12974-014-0161-z

**Published:** 2014-09-13

**Authors:** Petra Majerova, Monika Zilkova, Zuzana Kazmerova, Andrej Kovac, Kristina Paholikova, Branislav Kovacech, Norbert Zilka, Michal Novak

**Affiliations:** Institute of Neuroimmunology, Slovak Academy of Sciences, AD Centre, Dubravska cesta 9, 845 10 Bratislava, Slovak Republic; Axon Neuroscience SE, Dvorakovo nabrezie 10, 811 02 Bratislava, Slovak Republic

**Keywords:** Alzheimer’s disease, Extracellular tau, Tau oligomers, Neuroinflammation, Microglia, Macrophages

## Abstract

**Background:**

Abnormal misfolded tau protein is a driving force of neurofibrillary degeneration in Alzheimer’s disease. It has been shown that tau oligomers play a crucial role in the formation of intracellular neurofibrillary tangles. They are intermediates between soluble tau monomers and insoluble tau filaments and are suspected contributors to disease pathogenesis. Oligomeric tau can be released into the extracellular space and spread throughout the brain. This finding opens the question of whether brain macrophages or blood monocytes have the potential to phagocytose extracellular oligomeric tau.

**Methods:**

We have used stable rat primary microglial cells, rat peripheral monocytes-derived macrophages, BV2 microglial and TIB67 macrophage immortalized cell lines that were challenged by tau oligomers prepared by an *in vitro* aggregation reaction. The efficiency of cells to phagocytose oligomeric protein was evaluated with confocal microscopy. The ability to degrade tau protein was analyzed by immunoblotting.

**Results:**

Confocal microscopy analyses showed that macrophages were significantly more efficient in phagocytosing oligomerized tau proteins than microglial cells. In contrast to macrophages, microglia are able to degrade the internalized oligomeric tau only after stimulation with lipopolysaccharide (LPS).

**Conclusions:**

Our data suggests that microglia may not be the principal phagocytic cells able to target extracellular oligomeric tau. We found that peripheral macrophages display a high potency for elimination of oligomeric tau and therefore could play an important role in the modulation of neurofibrillary pathology in Alzheimer’s disease.

**Electronic supplementary material:**

The online version of this article (doi:10.1186/s12974-014-0161-z) contains supplementary material, which is available to authorized users.

## Background

Neuroinflammation plays a key role in the modulation of the pathogenesis of neurodegenerative disorders such as Alzheimer’s disease (AD) and related tauopathies [1–3]. In AD, activated microglia may become neurotoxic through their increased expression of pro-inflammatory cytokines, which lead to extensive neuronal death and disease manifestation [4,5]. Prolonged activation of microglia might impair their lysosomal enzymatic complex and such ‘frustrated phagocytes’ are not able to remove AD pathological lesions [6,7]. Another study demonstrated that microglia could internalize Aβ plaques but they released it back into the medium without degradation [8].

It has been hypothesized that macrophages which infiltrate the brain could be more effective in the recognition and subsequent elimination of the pathological lesions than resident microglia [9]. Simard *et al*. [10] showed that circulating cells such as monocytes could be responsible for effective phagocytosis and degradation of amyloid plaques in transgenic AD mice. Furthermore, peripheral macrophages were found to be particularly efficient in phagocytosing synthetic Aβ peptides [11].

Distribution of activated microglia parallels that of tau deposits in human AD [12–16] and non-AD tauopathies such as tangle-predominant dementia, progressive supranuclear palsy, corticobasal degeneration and Pick’s disease [17–21]. Activated microglia have been frequently present in the proximity of neurofibrillary tangles at early and late stages of tangle formation, which suggests a close relationship between inflammatory response and tau neurofibrillary lesions [14]. The activation of microglia linked to tau deposition has been also observed in various tau transgenic models [22–27].

Several previous studies have demonstrated that extracellular tau proteins play an important role in AD neuroinflammation [28–30]. It was shown that the oligomeric tau that represents an early stage of tangle formation can be released into the extracellular space, where it behaves as a transmissible agent, spreading tau pathology throughout the brain in a ‘prion-like fashion’ [31–33].

These findings raise the question of whether brain macrophages or peripheral monocytes entering the brain have the potential to phagocytose extracellular oligomeric tau and thus eliminate its toxic function. Therefore we have investigated the ability of BV2 microglia, immortalized TIB67 macrophages cell lines, peripheral blood monocytes-derived macrophages and primary microglia to phagocytose and degrade tau oligomers. Misfolded truncated tau was used as a template for tau oligomerization. Truncated tau species was identified by two monoclonal antibodies (MN423 and DC11) in the human AD brain [34–36]. The truncated tau used in this study was previously shown to induce abnormal microtubular assembly *in vitro* [37] and extensive neurofibrillary degeneration *in vivo* [37,38].

In this study we found that peripheral blood monocyte-derived macrophages (PB-MoM) and immortalized TIB67 macrophages are more proficient in the phagocytosis and degradation of oligomerized truncated tau than primary microglial cells or immortalized BV2 microglia.

## Materials and methods

### Expression and purification of recombinant tau protein

Human truncated tau151-391 (numbering according to the longest human tau isoform Tau40) was expressed in *Escherichia coli* strain BL21(DE3) (Sigma-Aldrich, St. Louise, Missouri, United States) from a pET-17 expression vector and purified from bacterial lysates as described previously [39], except that the phosphocellulose step was omitted and size-exclusion chromatography was performed in PBS (137 mM NaCl, 2.7 mM KCl, 10 mM Na_2_HPO_4_, 2 mM KH_2_PO_4_, pH 7.4) (AppliChem GmbH, Darmstadt, Germany). Purified tau protein was stored in PBS in working aliquots at −70°C. The purity of tau protein was subsequently verified by gradient SDS gel electrophoresis (5 to 20% gel), Coomassie blue staining and Western blot analysis with DC25 antibody (AXON Neuroscience SE (Bratislava, Slovakia), recognizes residues 347–354 of the longest human tau isoform Tau40). The fluorescently tagged tau protein was prepared by labelling with Alexa Fluor 546 (Invitrogen, Carlsbad, California, United States) according to the manufacturer’s recommendations.

### Tau filament assembly and detection of tau oligomers

*In vitro* oligomerization of recombinant truncated tau protein (aa 151–391, 100 μM) was carried out using heparin (Sigma-Aldrich, St. Louis, Missouri, United States) as an inducer at a final concentration of 25 μM in PBS (137 mM NaCl, 2.7 mM KCl, 10 mM Na_2_HPO_4_, 2 mM KH_2_PO_4_, pH 7.4) The reaction was performed overnight (for at least 12 hours) at 37°C. After incubation, tau oligomers were collected by centrifugation at 100,000 × g for 1 hour at room temperature and the pellet was re-suspended in PBS and sonicated for 5 seconds at 20% power output using an MS72 probe of a Bandelin Sonopuls Sonifier (Bandelin, Berlin, Germany). Subsequently, 1 μM aliquots were stored at -70°C. The oligomerization of the tau protein was verified by SDS gel electrophoresis, quantitative thioflavin T (ThT) fluorescence spectroscopy with excitation at 450 nm and emission at 510 nm, and by electron microscopy.

### Transmission electron microscopy

For morphological examination of in vitro oligomerized tau by electron microscopy the oligomers collected by centrifugation were dissolved in pure water (Merck Millipore, Darmstadt, Germany) and placed on carbon-coated 400 mesh copper grids (Christine Gröpl, Austria) for 2 minutes. Grids were washed with pure water for 2 minutes and the tau oligomers were negatively stained with 2% uranyl acetate for 1 minute (Sigma-Aldrich, St. Louis, Missouri, United States). The stained grids were immediately analyzed using an FEI Morgagni 268 electron microscope (Czech Republic).

### Cell cultivation

Mouse microglial BV2 cells (C57BL/6, purchased from ICLC, Modena, Italy) and mouse macrophages J774A.1 (TIB67™, purchased from ATCC™ (American Type Culture Collection (ATCC), La Jolla, California, United States) were cultivated in Dulbecco’s Modified Eagle’s Medium (DMEM, PAA laboratories GmbH, Colbe, Germany) containing 10% Fetal calf serum (FCS) (Invitrogen, Carlsbad, California, United States), and 2 mM L-glutamine (PAA laboratories GmbH, Colbe, Germany) at 37°C and 5% CO_2_. The medium was changed twice a week.

### Primary microglial culture

Cerebral cortices of newborn Sprague Dawley rats (Institute of Neuroimmunology, Bratislava, Slovakia) (1 day old) were dissected by cervical dislocation, stripped of their meninges, and mechanically dissociated by repeated pipetting followed by passing through a nylon mesh. Cells were plated in 96-well plates and 75 cm^2^ flasks pre-coated with poly-L-lysine (10 mg/ml) and cultivated in DMEM containing 10% FCS and 2 mM L-glutamine (all from Life Technologies Invitrogen, Carlsbad, California, United States) at 37°C, 5% CO_2_ in a water-saturated atmosphere. The medium was changed twice a week. Mixed glial cultures reached confluence after 8 to 10 days *in vitro*. Confluent mixed glial cultures were subjected to mild trypsinization (0.05 to 0.12%) in the presence of 0.2 to 0.5 mM Ethylenediaminetetraacetic acid (EDTA). This resulted in the detachment of an intact layer of cells containing astrocytes, leaving undisturbed a population of firmly attached cells identified as more than 98% microglia [40]. The cells were maintained in astrocyte-conditioned medium and were used for experiments after 24 hours in culture. The purity of microglial cell cultures isolated by this procedure was 95% (CD11b/CD18 staining).

### Peripheral blood monocyte-derived macrophages (PB-MoM)

Rat blood monocytes were obtained from the peripheral blood of healthy Sprague Dawley rat males (5-months-old). Aseptically, 8 ml of Ficoll-Paque Plus (GE Healthcare, Uppsala, Sweden) were added into a clean centrifuge tube. A total of 4 ml of blood samples diluted with sterile PBS 1:1 were carefully layered on Ficoll-Paque Plus (GE Healthcare, Uppsala, Sweden) and centrifuged at 300 × g for 30 minutes at 25°C.

Using a sterile Pasteur pipette the lymphocyte layer was transferred into a clean centrifuge tube and resuspended in PBS. The cells were centrifuged at 300 × g for 15 minutes at 25°C. Isolated cells were cultivated in Roswell Park Memorial Institute (RPMI) 1640 medium containing 20% FCS and 2 mM L-glutamine for 2 days. Then the cells were differentiated into peripheral blood monocyte-derived macrophages (PB-MoM) with granulocyte colony-stimulating factor GM-CSF (100 ng/ml) for a further 6 days.

### Cell stimulation

One day prior to the challenge of the cells with latex beads or tau oligomers, cell culture medium was replaced with serum-free DMEM medium with L-glutamine. To stimulate the cells, lipopolysaccharide (LPS) from *E. coli* (Sigma-Aldrich, St. Louis, Missouri, United States) was added to the medium for a final concentration of 250 ng/ml for 24 hours. This concentration of LPS had minimal effect on cell viability (data not shown).

### Phagocytosis assay

TIB67 macrophages and BV2 microglia cells were plated on 6-well plates at a density of 5 × 10^3^ cells/cm^2^. They were cultivated in the presence (stimulated) or absence (non-stimulated) of LPS and subsequently challenged either with 1 μM oligomeric tau (labelled with Alexa Fluor 546) or with red fluorescent latex beads (2 μm, L3030, Sigma-Aldrich, St. Louis, Missouri, United States) for 2, 6 and 24 hours in DMEM medium supplemented with L-glutamine without serum. The experiments were performed in triplicate. The phagocytic activities of the cells were evaluated by *in vivo* cell imaging, flow cytometry and Western blot analysis.

### In vivo cell imaging

Cells were plated (at a density of 5 × 10^3^ cells/well) on Lab-tek chamber slides (Thermo Scientific, Rockford, Illinois, United States) pre-coated with poly-L-lysine (Sigma-Aldrich, St. Louis, Missouri, United States) and cultivated for 24 hours in DMEM medium with serum. For visualization of live cells we used MitoTracker™ Green FM green-fluorescent mitochondrial stain (final concentration of 200 nM, Invitrogen, Carlsbad, California, United States). Staining was done for 30 minutes under normal growth conditions. After staining the solution was replaced with fresh pre-warmed Hank’s solution (Sigma-Aldrich, St. Louise, Missouri, United States and cells were examined by LSM 710 confocal microscope (Zeiss, Jena, Germany). At least 100 cells per chamber were analyzed for quantification, totalling ten chambers per experiment.

### Western blot analysis

Cells were plated at a density of 5 × 10^3^ cells/cm^2^ in a 6-well plates and cultivated with 1 μM oligomeric tau protein for 2, 6 and 24 hours. Before cell lysis, the cells were incubated with 0.05% trypsin for 3 minutes at 37°C to remove tau bound to the cell surface, the residual trypsin activity was blocked with 1% bovine serum albumin (BSA) in PBS for 30 minutes at room temperature and then the cells were harvested into lysis buffer (200 mM Tris, pH 7.4, 150 mM NaCl, 1 mM EDTA, 1 mM Na_3_VO_4_, 20 mM NaF, 0.5% Triton X-100, 1 × protease inhibitors complete EDTA-free from Roche, Mannheim, Germany). Total protein concentration of prepared cell extracts was measured by Bio-Rad protein assay (Bio-Rad laboratories GmbH, Colbe**,** Germany). A total of 25 μg of the proteins was separated onto 12% SDS-polyacrylamide gels and transferred to nitrocellulose membrane in 10 mM N-cyclohexyl-3-aminopropanesulfonic acid (CAPS, pH 11, Roth, Karlsruhe**,** Germany). The membranes were blocked in 5% milk in Tris-buffered saline/Tween 20 (Sigma-Aldrich, St. Louis, Missouri, United States) (TBS-T, 137 mM NaCl, 20 mM Tris-base, pH 7.4, 0.1% Tween 20) for 1 hour and incubated with primary antibody (DC25 antibody) overnight at 4°C. Membranes were incubated with horseradish peroxidase (HRP)-conjugated secondary antibody in TBS-T (1:3000, Dako, Glostrup, Denmark) for 1 hour at room temperature. Immunoreactive tau proteins were detected by chemiluminescence (SuperSignal West Pico Chemiluminescent Substrate, Thermo Scientific, Pittsburgh, United States) and the signals were digitized by Image Reader LAS-3000 (FUJIFILM, Bratislava, Slovakia).

### Data analysis and statistics

Each experiment was repeated at least three times. Data are presented as mean ± standard error of the mean (SEM). Friedman’s repeated measures one-way analysis of variance (ANOVA) on ranks and Dunn’s method for multiple comparisons were used. Student’s t-test was used for assessment of the difference between groups. Statistical significance was defined as *P* <0.05.

## Results

### Characteristic features of tau oligomers

**Figure 1:**
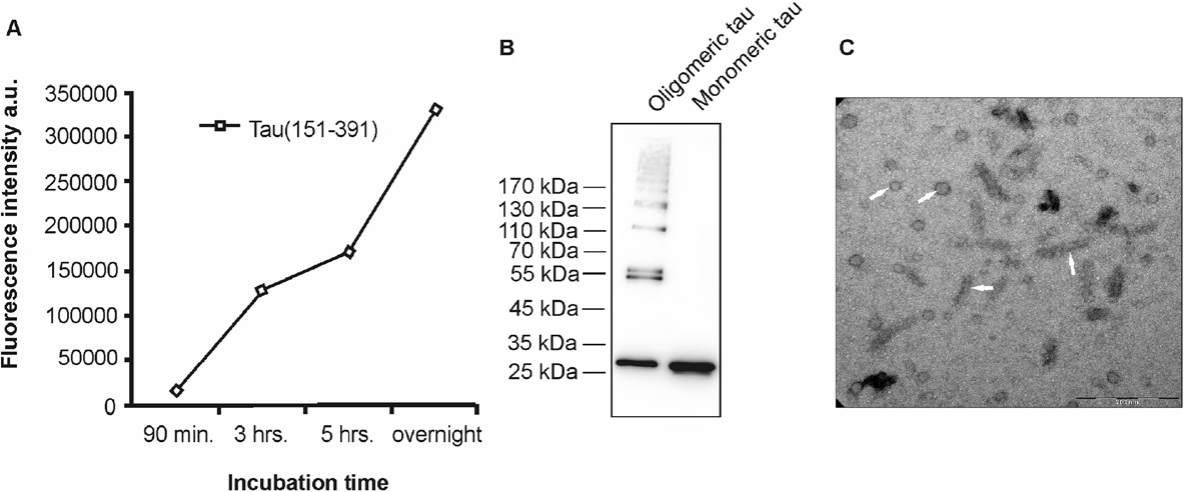
**Characterization of tau oligomers.** Oligomerized tau protein (tau151-391) was prepared by *in vitro* oligomerization reaction using polyanionic inducer heparin. Progress of tau oligomerization was monitored by thioflavin T fluorescence **(A)**. Neither tau nor heparin alone in the oligomerization reaction produced significant thioflavin T fluorescence over the time course of the experiment. Monomeric tau (25 kDa, apparent molecular weight on SDS-PAGE is 30 kDa) and oligomerized tau (45 to 170 kDA) was analyzed by Western blot and visualized by monoclonal antibody DC25 (epitope 347–354 of human tau isoform Tau40) **(B)**, which revealed multiple SDS-stable oligomeric species. Transmission electron microscopy **(C)** showed tau oligomers in the form of small round particles and short filaments (arrows). Scale bar represents 200 nm.

A characteristic feature of most neurodegenerative disorders is the accumulation of protein aggregates and pathologically modified protein forms. In order to analyze the phagocytic activities of microglia and macrophages we used insoluble tau oligomers generated *in vitro*. The oligomers were prepared from recombinant truncated tau (aa 151–391 of the longest human tau isoform Tau40) tagged with fluorescent dye Alexa Fluor 546 (Invitrogen, Carlsbad, California, United States). *In vitro* oligomerization of the recombinant truncated tau protein was induced by polyanionic aggregation inducer heparin. The reaction was allowed to proceed overnight at 37°C. To evaluate the progress of tau oligomerization we used thioflavin T fluorescence. Tau oligomerization increased in time with the highest fluorescent signal after 12 hours of incubation (Figure 1A).

We analyzed tau oligomers by Western blot analysis (detection with DC25 antibody, amino acids 347–354) and electron microscopy. Western blot showed higher molecular weights tau oligomers (45–170 kDa, Figure 1B).

Morphological examination of the oligomeric tau by transmission electron microscopy revealed the presence of small circular oligomers with diameters ranging from 10 to 30 nm and short filaments up to 120 nm long with a width of 10 to 15 nm (Figure 1C), similar to those described by Friedhoff *et al*. [41].

### Immortalized TIB67 macrophages are more effective in phagocytosis of tau oligomers than BV2 microglia cell line

To evaluate the mechanism of elimination of extracellular oligomeric tau present in the brains of tauopathy patients, we analyzed phagocytic activity of BV2 mouse microglia cell line, a widely used valid model of microglial cells [42,43], and monocytic/macrophage cell line J774A.1 (ATCC™ TIB67™) as a surrogate for primary macrophages [44]. Each experiment consisted of three replicates of cultures to confirm our findings. Three independent batches of cultures (BV2 and TIB67) or two or three independent isolations of either primary microglia or monocytes-derived macrophages were used (Additional file 1: Table S1).

The phagocytic study investigated four parameters: 1) phagocytic capacity (non-activated and activated) of the phagocytes against particulate matter, evaluated by standardized homogeneous particles, 2) phagocytic capacity (non-activated and activated) against abnormal oligomerized tau proteins, 3) temporal characterization of the phagocytic potency and 4) potency to degrade the internalized abnormal tau proteins. The first three parameters were analyzed using *in vivo* cell imaging and flow cytometry. The tau degradation activities were evaluated by Western blotting.

Phagocytic activity largely depends on the particle size. Several studies reported that maximal phagocytosis was observed for particle size with a diameter of about 2 μm and that phagocytosis decreased with increasing particle size [45]. Therefore, we characterized the phagocytic capacity of microglia and macrophages with standard red fluorescent latex beads with a diameter of 2 μm in activated (by LPS) and non-activated states. The process of phagocytosis was monitored by live fluorescent microscopy over the period of 24 hours at three independent time points (2, 6 and 24 hours). Individual cells were visualized by mitochondrial staining with MitoTracker™ Green FM.

**Figure 2:**
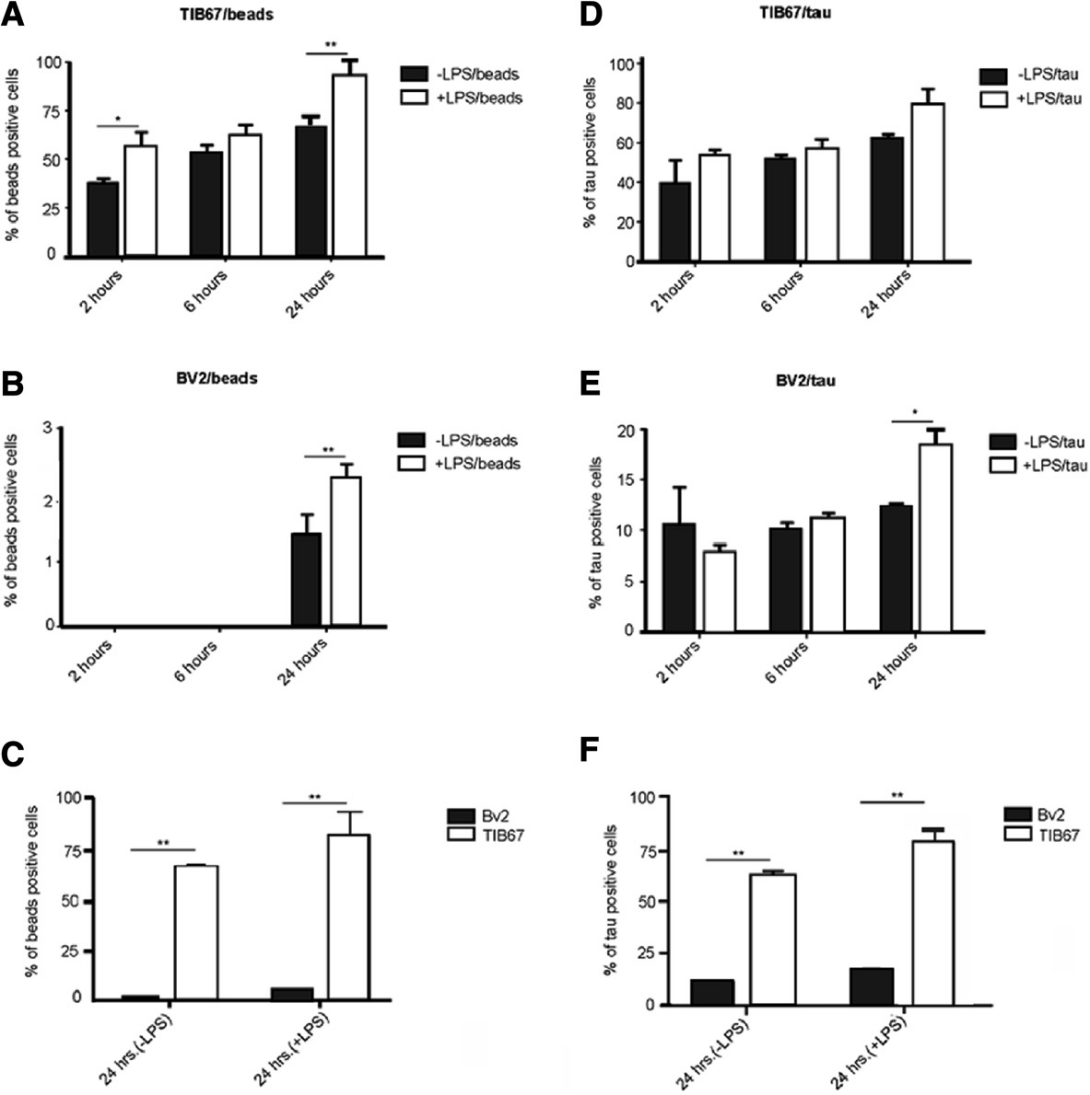
**Comparative immunofluorescent analysis uncovered the higher potency of TIB67 macrophages phagocytose tau oligomers comparing to BV2 microglia.** Live phagocytes were labelled with MitoTracker™ Green FM green-fluorescent mitochondrial stain and then challenged with red fluorescent latex beads or with oligomeric tau labelled with red Alexa Fluor 546. The panels show quantification data of TIB67 macrophages **(A)**, BV2 microglia **(B)** engulfing red fluorescent beads in the presence or absence of LPS treatment. **(C)** Quantitative comparison of TIB67 and BV2 phagocytosis of latex beads after 24 hours**.** Quantification of TIB67 macrophages **(D)** and BV2 microglia **(E)** phagocytosing tau oligomers in the presence or absence of LPS treatment. **(F)** Quantitative comparison of TIB67 and BV2 primary microglia cell phagocytosis of tau oligomers after 24 hours. The experiments were run in triplicates. Data are presented as mean ± standard error of the mean (SEM). Statistical significance was defined as *P* <0.05 (*) or *P* <0.01 (**). Abbreviation: P, p-value.

The activity of TIB67 in internalizing latex beads did not significantly differ over time, although it showed tendency towards increasing activity (Figure 2A, non-activated cells/–LPS: *P* = 0.112; activated cells/+LPS: *P* = 0.103). We quantified the percentage of latex beads-positive TIB67 macrophages after 2 hours (−LPS: 40% ± 2.1% versus + LPS: 56% ± 1.9%; *P* = 0.012), after 6 hours (−LPS: 54% ± 1.4% versus + LPS: 66% ± 0.7%; *P* = 0.25) and after 24 hours (−LPS: 69% ± 3% versus + LPS: 88% ± 1.6%; *P* = 0.01). Importantly, LPS treatment increased cell phagocytic activity with statistical significance at 2 and 24 hours (Figure 2A).

Compared to TIB67 macrophages, BV2 microglia cells showed decreased potency in phagocytosis of the latex beads. No beads-positive BV2 microglia cells (stimulated with LPS or not) were observed after 2 and 6 hours. The ability of BV2 microglia to engulf latex beads significantly increased during 24 hours both under normal conditions and after stimulation with LPS (−LPS: *P* = 0.0046; +LPS: *P* = 0.0001), but even then it remained negligible in comparison to macrophages. As was the case for macrophages, statistical analysis revealed that LPS treatment significantly increased their phagocytic activity only after 24 hours (Figure 2B, +LPS: 2.6% ± 0.3% versus –LPS: 1.3% ± 0.5%; *P* = 0.0015).

Thus, the overall quantification showed that the ability of macrophages to phagocytose particulate matter (exemplified by the latex beads) was higher compared to BV2 cells (Figure 2C). We found that after 24 hours 69% (±0.03%) of macrophages contained engulfed latex beads, compared to only 1.3% (±0.5%) of BV2 microglia (Figure 2C, *P* = 0.003). After LPS stimulation we detected 88% (±1.6%) beads-positive TIB67 macrophages but only 2.6% (±0.3%) beads-positive BV2 microglia (Figure 2C, *P* = 0.008).

In order to characterize the tauopathy-relevant phagocytic activity of the two types of phagocytes, we challenged the cultivated cells with oligomerized tau. The ability of TIB67 macrophages to internalize tau oligomers appeared very similar to their activity towards latex beads. The quantification of the percentage of oligomerized tau-positive macrophages showed that after 2 hours more than one third of all cells contained internalized tau oligomers (Figure 2D, 39% ± 2.3%), which increased over time to almost two thirds after 24 hours (Figure 2D, 6 hours: 51% ± 1.1%; 24 hours: 62% ± 3.1%). LPS-stimulated macrophages exhibited increased (albeit not significantly) phagocytic activity at all time points (Figure 2D, 2 hours: 54% ± 1.9%, –LPS versus + LPS *P* = 0.47; 6 hours: 60.3% ± 1.9%, −LPS versus + LPS *P* = 0.18; 24 hours: 79% ± 2.7%, −LPS versus + LPS *P* = 0.18). The ability of TIB67 macrophages to phagocytose tau oligomers significantly increased over time only after LPS treatment (Figure 2D, +LPS: *P* = 0.044; −LPS: *P* = 0.2). It is important to note that LPS stimulation had a rather minor effect on the phagocytosis of tau oligomers by macrophages.

Analysis of phagocytic activity of BV2 microglia revealed that less than 15% of all cells were able to internalize tau oligomers at any time point (Figure 2E, −LPS). LPS stimulation did not increase this phagocytic activity during the first 6 hours (2 hours: −LPS 10.3% ± 0.8%, +LPS 8.7% ± 0.09%, *P* = 0.45; 6 hours: −LPS 10.2% ± 0.5%, +LPS 11.4% ± 0.3%, *P* = 0.36) and only slightly after 24 hours (−LPS: 11.6% ± 0.6%; +LPS: 17.4% ± 0.3%, *P* = 0.011). This LPS-stimulated phagocytic activity significantly increased with time (*P* = 0.021).

Our comparative study revealed that the number of oligomerized tau-positive TIB67 macrophages was more than five times higher than the number of tau positive BV2 microglia cells (Figure 2F, 62% ± 3.1% versus 11.2% ± 0.6%; *P* = 0.003). Similar results were obtained after LPS stimulation (Figure 2F, 78.7% ± 2.7% versus 15.4% ± 0.3%, *P* = 0.004).

**Figure 3:**
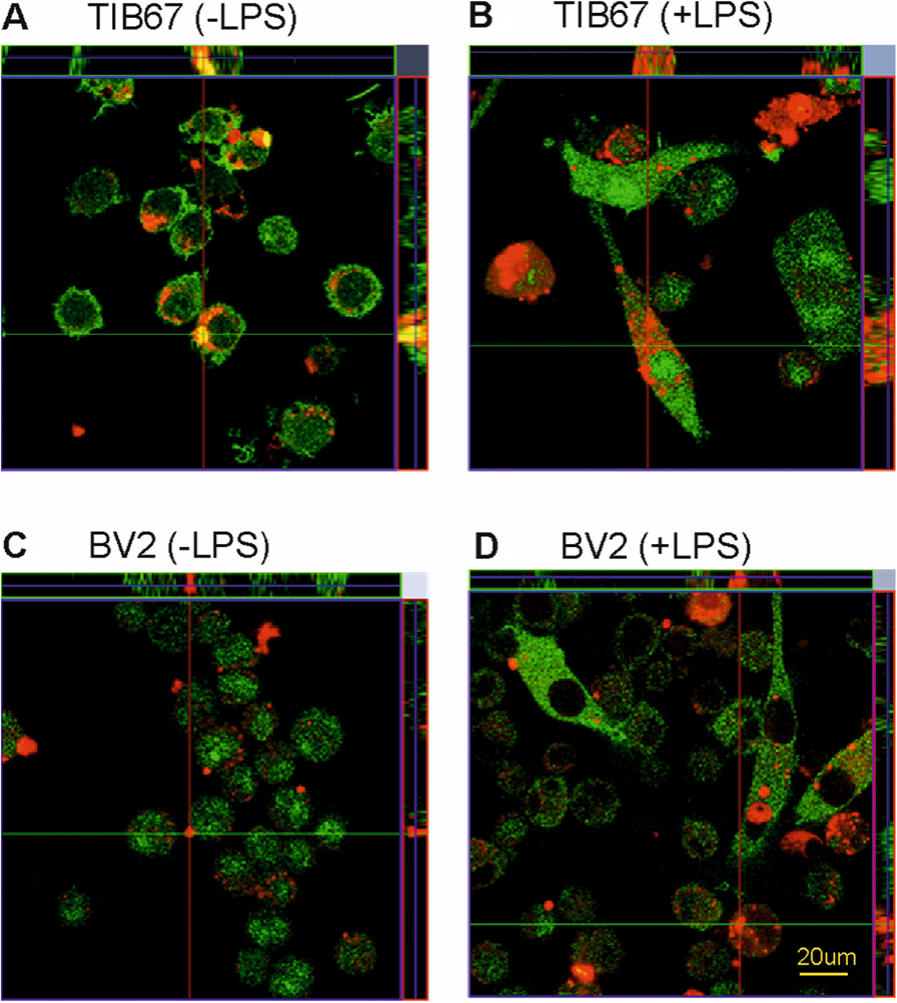
**Confocal imaging showing that TIB67 macrophages are more efficient in phagocytosis of tau oligomers than BV2 microglia.** Confocal cross-section analyses of cells show a higher number of tau oligomers engulfed by macrophages than by BV2 microglial cells. Micrographs show tau oligomers positive TIB67 macrophages non-treated **(A)** or treated with LPS **(B)**. Cross-sections show that tau is internalized within the cells. Tau oligomer positive BV2 cells without and with LPS stimulation are shown in panels **(C)** and **(D)**, respectively. LPS-stimulated microglia contain tau oligomers internalized, while non-stimulated cells have tau particles rather attached to the outside of the cells. Scale bar: 20 μm. Abbreviation: μm, micrometer.

We used the z-stacking function of the laser scanning confocal microscope to confirm that the tau oligomers are indeed internalized and not just attached to the outside of the cells (Figure 3). Confocal images of the phagocytes after 24 hours of incubation with tau oligomers showed that oligomerized tau (red) is localized within the cell bodies (green) of TIB67 macrophages (Figure 3A - without LPS stimulation, Figure 3B - with LPS stimulation). In the case of non-stimulated BV2 microglia, there is a smaller amount of tau oligomers associated with the cells and it seems rather attached to the outside of the cells (Figure 3C). On the other hand, LPS-stimulated microglia contain larger amounts of tau within the cells, although the amount is still smaller than that found in macrophages (Figure 3D). These observations support data coming from our quantitative study where we found that BV2 microglia are less potent in phagocytosing tau oligomers than TIB67 macrophages.

### Peripheral rat blood monocyte-derived macrophages are more effective in phagocytosis of tau oligomers than rat primary microglia

In order to show that our analysis is relevant to an *in vivo* situation, we tested the phagocytic activity of primary microglial cells and peripheral rat blood monocyte-derived macrophages (PB-MoM) as well.

**Figure 4:**
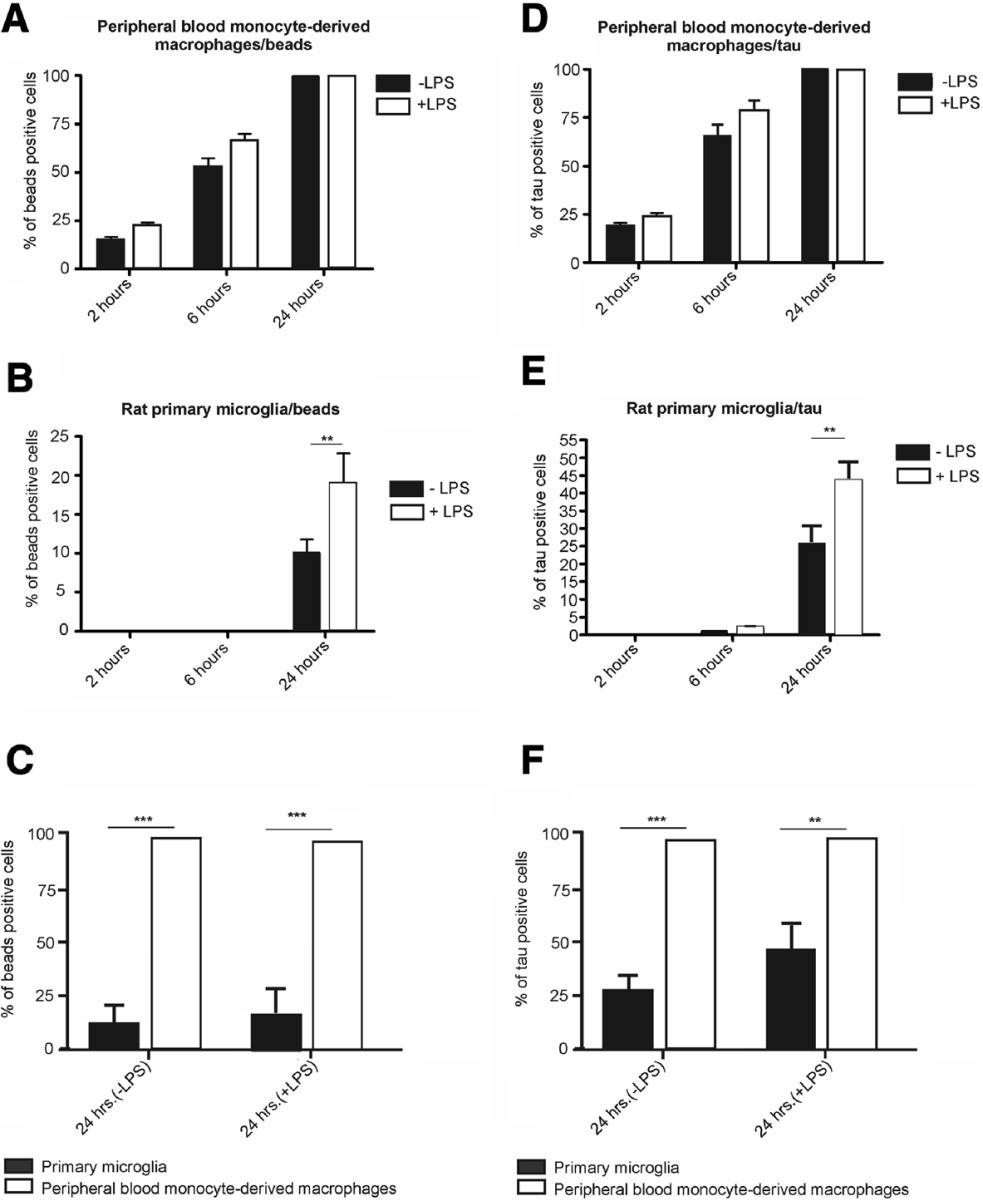
**Immunofluorescent study reveals a higher efficacy of peripheral blood-derived monocytes to phagocytose tau oligomers than primary microglia.** The panels show quantification data of PB-MoM macrophages **(A)** and primary microglia **(B)** engulfing oligomerized beads in the presence or absence of LPS treatment. **(C)** Quantitative comparison of PB-MoM macrophages and primary microglia phagocytic efficacy of beads oligomers after 24 hours**.** Quantification of PB-MoM macrophages **(D)** and primary microglia **(E)** phagocytosing tau oligomers in the presence or absence of LPS treatment. **(F)** Quantitative comparison of PB-MoM macrophages and primary microglia and primary microglia cells phagocytosis of tau oligomers after 24 hours. The experiments were run in triplicates. Data are presented as mean ± standard error of the mean (SEM). Statistical significance was defined as *P* <0.05 (*) or *P* <0.01 (**). Abbreviations: SEM, standard error of the mean; P, p-value; PB-MoM, Peripheral blood monocyte-derived macrophages.

The activity of PB-MoM in internalizing latex beads increased over time (Figure 4A, non-activated cells/–LPS: *P* = 0.0015; activated cells/+LPS: *P* = 0.0019). We quantified the percentage of latex beads-positive PB-MoM after 2 hours (−LPS: 15% ± 1.2% versus + LPS: 25% ± 1%; *P* = 0.052), after 6 hours (−LPS: 51% ± 1.4% versus + LPS: 68% ± 1%; *P* = 0.23) and after 24 hours (−LPS: 100% ± 0.3% versus + LPS: 100% ± 0.6%). Importantly, LPS treatment did not increase the cell phagocytic activity over time.

The potency of primary microglia to engulf latex beads increased during 24 hours both under normal conditions and after stimulation with LPS (Figure 4B, −LPS: *P* = 0.0002; +LPS: *P* = 0.001). After 24 hours, the phagocytic activity of primary microglia did not reach the activity of peripheral macrophages (Figure 4B, −LPS: 10.8% ± 0.6% versus + LPS: 19.3% ± 1%). Strikingly, LPS treatment significantly increased the cell phagocytic activity after 24 hours (*P* = 0.0035).

We found that after 24 hours, 100% of peripheral rat blood monocyte-derived macrophages contained engulfed latex beads, while only 10.8% (±0.5%) of primary microglia showed such inclusions (Figure 4C, *P* = 0.001). After LPS stimulation we detected 100% beads-positive macrophages, but only 19.3% beads-positive primary microglia (Figure 4C, *P* = 0.0012).

The analysis of the phagocytosis of tau oligomers by peripheral rat blood monocyte-derived macrophages and primary microglia revealed interesting differences. PB-MoM exhibited an extremely high ability to phagocytose oligomerized tau. The quantification of the percentage of oligomerized tau-positive PB-MoM increased over time (Figure 4D, 2 hours: 19% ± 1.3%; 6 hours: 69% ± 2.1%; 24 hours: 100% ± 0.1%; *P* = 0.011). LPS-stimulated macrophages exhibited increased (but not significantly) phagocytic activity at all time points (Figure 4D, 2 hours: 24% ± 0.9%; 6 hours: 77.3% ± 1.8%; 24 hours: 100% ± 0.7%). Similarly to TIB67 cells, LPS stimulation had no effect on phagocytosis of tau oligomers by peripheral rat blood monocyte-derived macrophages.

Primary microglial cells displayed limited phagocytic activity during the 6-hour incubation time, even after LPS stimulation (Figure 2E, 2 hours: −LPS 0.3% ± 0.08%, +LPS 0.42% ± 0.009%, *P* = 0.65; 6 hours: −LPS 1.8% ± 0.05%, +LPS 2.4% ± 0.03%, *P* = 0.36). The activity increased after LPS stimulation only after 24 hours (−LPS: 26.4% ± 0.3%, +LPS: 42.6% ± 0.6%, *P* = 0.01).

Thus, the overall quantification showed that the ability of peripheral rat blood monocyte-derived macrophages to phagocytose oligomerized tau was higher compared to primary microglia (Figure 4F). We found that after 24 hours 100% of PB-MoM macrophages contained engulfed oligomerized tau, compared to only 26.4% of primary microglia (Figure 2F, *P* = 0.001). After LPS stimulation we detected 100% tau positive PB-MoM macrophages and 42.6% tau-positive primary microglia cells (Figure 4F, *P* = 0.0014).

Our comparative study revealed that the number of oligomerized tau-positive PB-MoM macrophages was almost four times higher than the number of tau positive primary microglia cells in the absence of LPS, and two times higher after stimulation with LPS.

**Figure 5:**
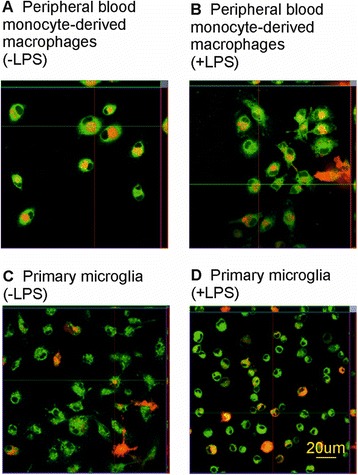
**Confocal study showed that peripheral blood-derived monocytes are more efficient in phagocytosis of tau oligomers than primary microglia.** Confocal cross-section analyses of cells showed higher number of tau oligomers engulfed by macrophages as by BV2 microglial cells. Micrographs show tau oligomers positive TIB67 macrophages non-treated **(A)** or treated with LPS **(B)**. The pictures demonstrate that almost all macrophages engulfed oligomeric tau. Primary microglia phagocytosing tau oligomers in the presence or absence of LPS treatment are shown in panels **(C)** and **(D)**. Primary cells showed higher ability to phagocyte tau oligomers after LPS stimulation. Scale bar: 20 μm. Abbreviations: μm, micrometer; LPS, lipopolysaccharide.

A confocal study revealed that PB-MoM are very effective in phagocytosis of tau oligomers regardless of LPS treatment (Figure 5A,B). In the case of non-stimulated primary microglia cells, there is a smaller amount of tau oligomers associated with the cells (Figure 5C), while LPS-stimulated microglia seems to be more effective in phagocytosis of tau oligomers than non-treated microglia (Figure 5D).

### Western blot analysis revealed that LPS stimulation increased degradation of tau oligomers by macrophages and microglia

**Figure 6:**
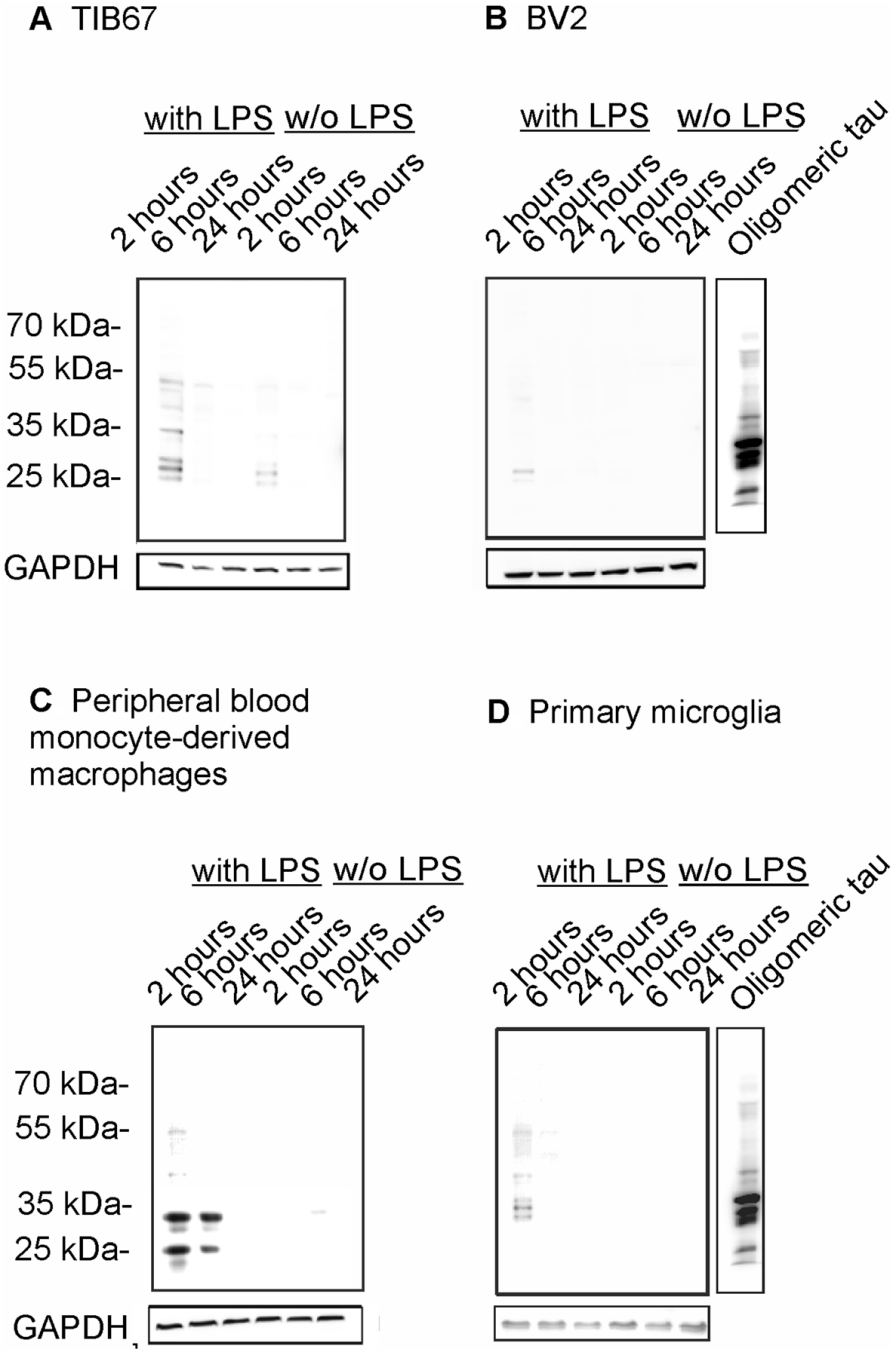
**Macrophages and microglia can degrade tau oligomers.** Western blot analyses of extracts from TIB67 macrophages **(A)**, BV2 microglia **(B)**, peripheral blood monocyte-derived macrophages **(C)** and primary microglia **(D)** challenged with oligomerized (misfolded) tau for 2, 6 and 24 hours showed that macrophages are more potent in degradation of tau oligomers than microglia. Microglia were able to phagocytose and degrade tau oligomers only after LPS stimulation. Western blots were developed with DC25 antibody. w/o, without. Abbreviations: w/o, without; LPS, lipopolysaccharide.

In order to evaluate the ability of macrophages to degrade phagocytosed tau oligomers we analyzed the presence of internalized tau by Western blot analysis. For total tau staining we used phosphorylation independent antibody DC25. The Western blot showed a decrease of oligomeric tau with time in the cell lysates from macrophages and microglia (Figure 6A,B,C,D, lane ‘2 hours’ compared to ‘6 hours’ and ‘24 hours’). Interestingly, TIB67 macrophage cells can phagocytose oligomeric tau regardless of LPS treatment, however the efficacy is higher after LPS stimulation (Figure 6A). Neither BV2 microglia nor primary microglia are able to degrade tau oligomers without LPS treatment (Figure 6B,D).

## Discussion

Alzheimer’s disease (AD) is a widespread progressive neurodegenerative disorder characterized by the presence of neurofibrillary tangles consisting of hyperphosphorylated and truncated tau proteins and extracellular senile plaques composed of amyloid-β [46–51]. Previous studies have indicated that neuroinflammation significantly modulates the pathogenesis of AD by increased activation of brain immune cells and their potency to phagocytose and enzymatically degrade pathologic lesions [52–54].

In early stages of AD, microglia activation becomes protective and delays the disease progression by effective clearance of pathological lesions [55–57], while in the later stages, microglia lose their protective function and release pro-inflammatory cytokines such as tumor necrosis factor-alpha (TNF-α) and interleukin-1beta (IL-1β) [4,5,58–60]. It was shown that microglia cells isolated from the aged brains exhibit increased release of cytokines as compared to those from the young brains, suggesting an elevated inflammatory state in aged microglia [61].

The issue of whether microglia are able to eliminate pathological lesions such as neurofibrillary tangles or senile plaques from AD brain still remains the matter of controversy. Wisniewski *et al*. [6] observed that human brain-resident microglia cells appeared in the close proximity of amyloid plaques but they did not contain β-amyloid fibrils in their lysosomal compartment. In contrast to this finding, some authors demonstrated that human microglia cells obtained from AD brains could engulf Aβ [52,62], however they could not degrade fibrillar Aβ [8,63]. Microglia with a defective lysosomal enzymatic complex have weak acidic lysosomes, which would decrease the activity of many proteases and could lead to the inability to degrade Aβ effectively [64]. On the other hand, it has been suggested that the acidic conditions in microglial endosomes and lysosomes can promote assembly or growth of fibrillar Aβ plaques, which could then be released from the cells into the extracellular space [65–67].

It was shown that tau oligomers were significantly elevated in the AD brain, preceded the tangle formation and had contributed to the progression of tau neurodegeneration [31,32]. In our study, we used recombinant truncated tau (aa 151-391/4R) derived from AD brains [37] to prepare tau oligomers. In order to analyze phagocytic activity of microglia we used immortalized mouse microglia cell line BV2 and primary microglial cells. Several research papers have demonstrated that BV2 cells can be used as alternative model system for primary microglia cultures for experiments examining brain inflammation, and faithfully mimic behaviors of primary microglia cells [68], as well as exhibit a robust phagocytic response [69–71]. Our results show that neither primary microglial cells nor BV2 microglia were able to effectively phagocytose oligomeric tau when compared with macrophages. However, after activation with LPS, the phagocytic activity of both primary microglial cells and BV2 cells increased. Previously, Cooper *et al*. [72] found that LPS could increase the phagocytic activity of microglia via increased Fc receptor capacity. Further, phagocytosis-related events, such as enhanced hexose monophosphate shunt activity, H_2_O_2_ formation and nitroblue tetrazolium reduction could also be stimulated by LPS [72]. We can hypothesize that LPS stimulation can activate molecular pathways involved in phagocytosis of tau oligomers.

Our Western blot analyses showed that LPS can stimulate degradation of oligomeric tau. Treatment with inflammatory agents such as LPS probably acidifies microglial lysosomes and thus increases degradation of oligomerized tau protein. It has been shown that stimulation by LPS increased the expression of CD68 that is responsible for lysosomal activity [73]. This indicates that acute inflammation can improve the microglial ability to phagocytose oligomeric tau.

The question of whether blood-borne leukocytes can penetrate into the AD brain yielded contradictory results. Some authors proposed that the chronic inflammatory response implicated in AD was provided almost exclusively by resident CNS cells without any apparent influx of leukocytes from the blood [3,74]. Others have demonstrated that hematopoietic cells can enter the AD brain [75–78] or rodent model brain [26,79,80]. Some animal studies using bone marrow transplant with green fluorescent protein-expressing myeloid cells suggest that monocytes are recruited to the brain parenchyma, where they evolve morphologically and functionally into microglia [81]. It was also shown that no brain resident microglia cells but circulating peripheral monocytes could be responsible for the phagocytosis and degradation of senile plaques in rat and human brain [81]. Fiala *et al*. [82] also showed that peripheral macrophages are highly capable of phagocytosing synthetic Aβ peptides. On the other hand, the presence of β-amyloid fibrils was observed in lysosomes of peripheral macrophages that infiltrated the brains of AD patients after stroke.

Here we show that macrophages are more effective in the phagocytosis of tau oligomers, the main component of neurofibrillary tangles in AD, than microglial cells. Our finding that macrophages succeed in the degradation of tau oligomers has provided insight into the possibility of utilizing them for targeted cell therapy for AD.

## Conclusions

Our data suggests that similar to the case with Aβ, microglia are not efficient enough to remove extracellular oligomeric tau. However we can conclude that macrophages display potential in the elimination of oligomeric tau from the AD brain.

## Additional file

Additional file 1: Table S1. Raw data used for statistical analysis.

## Abbreviations

Aa: Amino acids
Aβ: Amyloid-beta
AD: Alzheimer disease
BBB: Blood-brain barrier
CNS: Central nervous system
IL-1β: Interleukin-1beta
LPS: Lipopolysaccharide
MHC-II: Major histocompatibility complex class II
NFT: Neurofibrillary tangle
ThT: Thioflavin T
TNF-α: Tumor necrosis factor-alpha
SEM: standard error of the mean
P: p-value
w/o: without
um: micrometer
PB-MoM: Peripheral blood monocyte-derived macrophages
