# Supplementary material for: Microglia display modest phagocytic capacity for extracellular tau oligomers
Source: J Neuroinflammation. 2014 Sep 13;11:161. doi: 10.1186/s12974-014-0161-z (PMC4172893; doi:10.1186/s12974-014-0161-z)
Supplement: Additional file 1: Table S1. — Raw data used for statistical analysis. [file 12974_2014_161_MOESM1_ESM.docx]

| **BV2** | | | | | | | | | | | | | |
| --- | --- | --- | --- | --- | --- | --- | --- | --- | --- | --- | --- | --- | --- |
|  | **Beads (-LPS)**  **(%)** | | | **Beads (+LPS)**  **(%)** | | | **Tau (-LPS)**  **(%)** | | | | **Tau (+LPS)**  **(%)** | | |
|  | **2 hrs.** | **6 hrs.** | **24 hrs.** | **2 hrs.** | **6 hrs.** | **24 hrs.** | **2 hrs.** | | **6 hrs.** | **24 hrs.** | **2 hrs.** | **6 hrs.** | **24 hrs.** |
| **1. Experiment** | 0 | 0 | 1 | 0 | 0 | 2,3 | 9,8 | | 9,6 | 12,1 | 8 | 11 | 17 |
|  | 0 | 0 | 1,6 | 0 | 0 | 2,6 | 9,2 | | 10 | 11,2 | 7,3 | 11,2 | 17,9 |
|  | 0 | 0 | 0,8 | 0 | 0 | 3 | 10 | | 10,5 | 11,8 | 7 | 11,8 | 17,7 |
| **2. Experiment** | 0 | 0 | 1,5 | 0 | 0 | 3,3 | 10,2 | | 10,8 | 10,8 | 9 | 10 | 17,3 |
|  | 0 | 0 | 1,5 | 0 | 0 | 2,8 | 11 | | 11 | 11 | 9,2 | 11,7 | 17,7 |
|  | 0 | 0 | 1,2 | 0 | 0 | 3 | 12,2 | | 10,2 | 12,3 | 8,9 | 11,9 | 17,8 |
| **3. Experiment** | 0 | 0 | 1 | 0 | 0 | 2 | 10 | | 9 | 11,7 | 10 | 11,7 | 17,6 |
|  | 0 | 0 | 1,4 | 0 | 0 | 2,2 | 9,1 | | 10,4 | 11,5 | 10,9 | 11,5 | 16,6 |
|  | 0 | 0 | 1,8 | 0 | 0 | 2,5 | 11 | | 10,3 | 11,9 | 8 | 11,9 | 16,9 |
| **Average** | **0** | **0** | **1,3** | 0 | 0 | **2,6** | **10,3** | | **10,2** | **11,6** | **8,7** | **11,4** | **17,4** |
| **TIB67** | | | | | | | | | | | | | |
|  | **Beads (-LPS) (%)** | | | **Beads (+LPS) (%)** | | | **Tau (-LPS) (%)** | | | | **Tau (+LPS) (%)** | | |
|  | **2 hrs.** | **6 hrs.** | **24 hrs.** | **2 hrs.** | **6 hrs.** | **24 hrs.** | **2 hrs.** | **6 hrs.** | | **24 hrs.** | **2 hrs.** | **6 hrs.** | **24 hrs.** |
| **1. Experiment** | 41 | 54,6 | 68,8 | 55,7 | 66,1 | 86,6 | 40 | 50,9 | | 62 | 54 | 58 | 77,7 |
|  | 39,8 | 53,9 | 70,8 | 56 | 66,5 | 88,1 | 40,1 | 51 | | 62 | 52,8 | 59,2 | 80 |
|  | 40,2 | 53,3 | 65,6 | 56,3 | 66,2 | 90,3 | 39,7 | 51,6 | | 61,7 | 52 | 58,8 | 79,1 |
| **2. Experiment** | 40 | 54,1 | 64,9 | 56,4 | 67 | 88,7 | 39 | 50 | | 61 | 56,7 | 61,2 | 77,9 |
|  | 41,5 | 54,6 | 66,6 | 55,6 | 66,5 | 87,9 | 38,7 | 50,2 | | 64 | 55,9 | 58 | 80,9 |
|  | 39,2 | 54 | 71,4 | 55 | 66 | 87 | 39 | 51 | | 62 | 56 | 60 | 80 |
| **3. Experiment** | 39,9 | 53,2 | 70,8 | 56,6 | 65,8 | 88,1 | 38,4 | 51,6 | | 62,8 | 54,2 | 62,3 | 79 |
|  | 39 | 54,7 | 69,7 | 56,9 | 66,4 | 88 | 39 | 51,9 | | 62 | 53,9 | 63 | 76,9 |
|  | 40,3 | 53,8 | 72 | 55,9 | 65,3 | 87,5 | 38 | 51 | | 60,5 | 52 | 62 | 80 |
| **Average** | **40,1** | **54** | **69** | **56** | **66,2** | **88** | **39,1** | **51** | | **62** | **54,17** | **60,28** | **79,06** |

**Additional file 1: Table S1. Raw data used for statistical analysis.**

| **Primary microglia** | | | | | | | | | | | | |
| --- | --- | --- | --- | --- | --- | --- | --- | --- | --- | --- | --- | --- |
|  | **Beads (-LPS) (%)** | | | **Beads (+LPS) (%)** | | | **Tau (-LPS) (%)** | | | **Tau (+LPS) (%)** | | |
|  | **2 hrs.** | **6 hrs.** | **24 hrs.** | **2 hrs.** | **6 hrs.** | **24 hrs.** | **2 hrs.** | **6 hrs.** | **24 hrs.** | **2 hrs.** | **6 hrs.** | **24 hrs.** |
| **1. Experiment** | 0 | 0 | 10,9 | 0 | 0 | 19 | 0 | 2,0 | 27 | 0 | 2 | 41,9 |
|  | 0 | 0 | 11,6 | 0 | 0 | 19,2 | 0 | 2,0 | 26,3 | 0 | 3 | 42,8 |
|  | 0 | 0 | 10,8 | 0 | 0 | 19,3 | 0 | 2,3 | 26 | 0 | 3,3 | 42,6 |
| **2. Experiment** | 0 | 0 | 10,2 | 0 | 0 | 19,8 | 0,5 | 1,0 | 26,2 | 0,46 | 2,2 | 42,2 |
|  | 0 | 0 | 11,3 | 0 | 0 | 19 | 0,42 | 1,2 | 27,2 | 0,5 | 2,5 | 42,8 |
|  | 0 | 0 | 10,9 | 0 | 0 | 20,2 | 0,36 | 1,6 | 26,1 | 1 | 2,3 | 42,6 |
| **3. Experiment** | 0 | 0 | 10 | 0 | 0 | 19,2 | 0,4 | 2,1 | 26 | 0,2 | 2,1 | 42,9 |
|  | 0 | 0 | 10,7 | 0 | 0 | 19 | 0,2 | 1,9 | 26 | 0,6 | 2,2 | 42,9 |
|  | 0 | 0 | 10,9 | 0 | 0 | 19,6 | 0,9 | 1,9 | 27 | 1 | 2 | 43 |
| **Average** | **0** | **0** | **10,81** | **0** | **0** | **19,37** | **0,31** | **1,8** | **26,42** | **0,42** | **2,40** | **42,63** |
| **Monocyte-derived macrophages** | | | | | | | | | | | | |
|  | **Beads (-LPS) (%)** | | | **Beads (+LPS) (%)** | | | **Tau (-LPS) (%)** | | | **Tau (+LPS) (%)** | | |
|  | **2 hrs.** | **6 hrs.** | **24 hrs.** | **2 hrs.** | **6 hrs.** | **24 hrs.** | **2 hrs.** | **6 hrs.** | **24 hrs.** | **2 hrs.** | **6 hrs.** | **24 hrs.** |
| **1. Experiment** | 15,1 | 51 | 100 | 25,7 | 67,7 | 100 | 19 | 70 | 100 | 23,9 | 77 | 100 |
|  | 14,4 | 50,2 | 100 | 24,1 | 68 | 100 | 20,1 | 71 | 100 | 25 | 76,9 | 100 |
|  | 15,9 | 50,3 | 100 | 24,8 | 68,2 | 100 | 19,9 | 71,8 | 100 | 24,9 | 77,5 | 100 |
| **2. Experiment** | 15,8 | 51 | 100 | 25,7 | 67 | 100 | 19,6 | 69 | 100 | 24,1 | 77,3 | 100 |
|  | 15,2 | 51,6 | 100 | 24,3 | 67,4 | 100 | 19,6 | 69 | 99,9 | 24,8 | 77,6 | 100 |
|  | 14,3 | 51,3 | 100 | 25,3 | 68,2 | 100 | 19 | 67,7 | 99,8 | 24 | 77,8 | 100 |
| **Average** | **15,1** | **50,9** | **100** | **25,0** | **67,8** | **100** | **19,5** | **69,8** | **99,95** | **24,5** | **77,4** | **100** |
